# Appropriate follow-up period for odontogenic keratocyst: a retrospective study

**DOI:** 10.1186/s40902-021-00301-x

**Published:** 2021-07-01

**Authors:** Hwi-Dong Jung, Jung-Hwan Lim, Hyung Jun Kim, Woong Nam, In-Ho Cha

**Affiliations:** 1 Department of Oral and Maxillofacial Surgery, Oral Science Research Center, Yonsei University College of Dentistry, 50 Yonsei-ro, Seodaemoon-gu, Seoul, 03722 Republic of Korea; 2 Department of Oral and Maxillofacial Surgery, Yongin Severance Hospital, Yonsei University College of Dentistry, Seoul, Republic of Korea; 3 Oral Cancer Research Institute, Yonsei University College of Dentistry, Seoul, Republic of Korea

**Keywords:** Odontogenic kerotocyst, OKC, Keratocystic odontogenic tumor, KCOT, Recurrence, Recurrence of OKC

## Abstract

**Purpose:**

The aim of this study was to conduct epidemiologic investigations on the pattern of the lesion and differences between treatment modalities in terms of recurrence by reviewing follow-up records to form a basis for planning patient follow-up visits.

**Materials and methods:**

In this retrospective, single-center cohort study, 266 patients diagnosed with odontogenic keratocyst between 1993 and 2013 were included. Medical records and radiographic images were analyzed for age distribution, occurrence site and size, treatment modalities, and recurrence.

**Results:**

The average age at first diagnosis was 33.1 years, and the male to female ratio was 1.33:1.00. The highest rate of incidence was in the third decade followed by the fourth, second, and fifth decades. The incidence in the maxilla was 34%, and 66% in the mandible. Mandibular ramus was most commonly involved. Lesions between 3 and 6 crowns were the most common, and the rate of recurrence increased with size. Enucleation after decompression had higher rate of recurrence (35.8%) than enucleation (27.1%), but there was no statistical significance.

**Conclusion:**

The recurrence of odontogenic kerotocyst (OKC) was significantly associated with large size, multilocular form, and surgical procedure. A 10-year follow-up period is recommended to determine any recurrence of OKC.

**Supplementary Information:**

The online version contains supplementary material available at 10.1186/s40902-021-00301-x.

## Introduction

Odontogenic keratocyst (OKC) is a developmental cyst, which is thought to originate from the dental lamina [1]. It is most commonly encountered between the second and fifth decades, and affects the posterior mandibular most frequently [2–4]. OKC was classified as a tumor by the World Health Organization from 2005 to 2017 because of its high relapse rate, invasiveness, presence of epithelial dysplasia, and the recognition of gene mutations in tumor markers such as PTCH1 and Ki-67 [5–7]. In 2017, the new WHO/IARC classification reclassified OKC back into the cystic category because the evidence for its classification as a tumor was insufficient [8]. OKCs may be asymptomatic and found incidentally on X-rays, which makes early detection difficult [9]. While swelling is the most common complaint, symptoms related to infection or pathologic fracture caused by bone expansion may arise rarely.

The recurrence rate of OKC has been reported to be as high as 58.3% [4]. The reasons for the high recurrence rate are the following: the cystic epithelium is thin and has lower tensile strength than other maxillofacial tumors [10], it may be difficult to completely remove the tumor in toto [11, 12], and satellite cysts are often present [13]. Annual radiographic review with long-term clinical follow-up has been recommended. Although many researchers have reported the above features, it is advantageous to have more data to better understand the OKC.

The aim of this study was to conduct epidemiologic investigations on the pattern of the lesion and differences between treatment modalities in terms of recurrence by reviewing long-term follow-up records from 1993 to 2013. This could be the basis for planning patient follow-up visits.

## Materials and methods

This retrospective study conforms to the Declaration of Helsinki on medical protocols and ethics, and was received informed consents waiver approval from the regional Ethical Review Board of Yonsei University Dental Hospital Institutional Review Board (IRB 2-2014-0031).

A retrospective cohort study was planned using data of subjects who were diagnosed with OKC at the Department of Oral and Maxillofacial Surgery, Yonsei University College of Dentistry between 1993 and 2013. At first, a total 358 patients were included in this cohort. The exclusion criteria were as follows: insufficient medical records for classification of the lesion, or the method of operation; recurrence after previous surgery at another hospital; OKC associated with basal cell nevus syndrome; or orthokeratinized OKC following pathologic confirmation. Finally, applying the above criteria, this research was conducted using data of 266 patients. However, there were multiple lesions in 5 patients. Thus, a total 274 lesions were included.

**Fig. 1:**
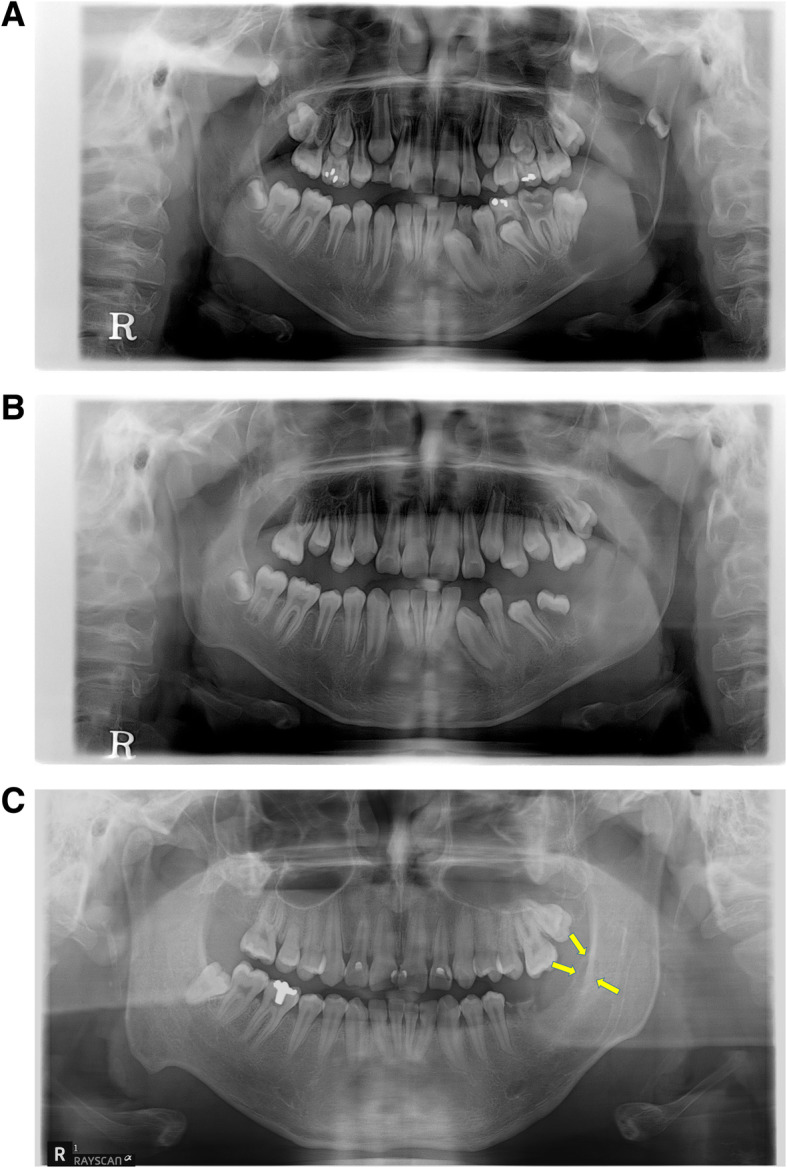
The example for recurrence case of OKC. **a** Pre-op. panoramic view. **b** Post-op. panoramic view. **c** Recurrence was observed after the initial enucleation surgery 10 years postoperatively

A total of 266 patients and 274 lesions were classified according to age, sex, location, and size of the lesion. Then, the recurrence according to the location and method of treatment was investigated (Fig. 1). The sites of occurrence and recurrence were designated on the maxilla and mandible considering the following points. The maxilla was divided into the anterior and posterior regions by the canine. The mandible was divided into three regions, as anterior, posterior, and the ramus by the location of the canine and the second molar. The size of the lesion was classified as a lesion less than 3 crowns, between 3 and 6 crowns, and larger than 6 crowns based on the mesiodistal width of the mandibular first molar crown in the panoramic radiograph. According to the radiographic findings, the lesion was classified as unilocular or multilocular lesion. In addition, according to the treatment method, it was classified as enucleation, enucleation following decompression, or en bloc excision.

### Statistical analysis

The statistical analysis was performed using SPSS version 22.0 software (SPSS, Inc. Chicago, IL). Cross-analysis was performed to confirm the difference in the recurrence rate of lesions according to the classification criteria described above. Chi-square test, Fisher’s exact test, and linear by linear association were used as statistical analytical methods. A life table was used to confirm the tendency of recurrence over time.

## Results

**Fig. 2:**
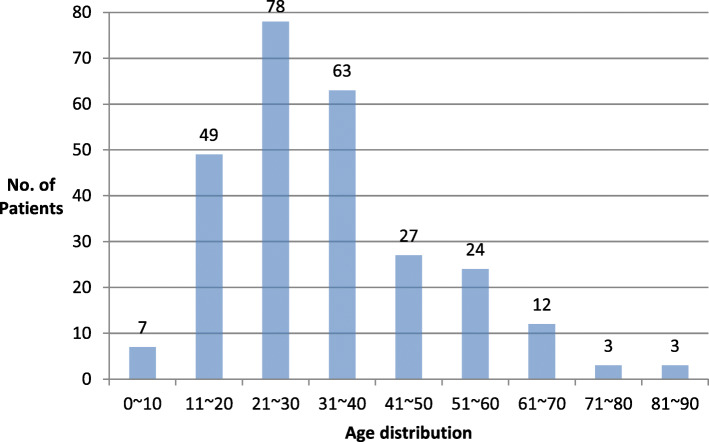
Age distribution in patients with odontogenic keratocyst

Of the 266 patients, 152 (57%) were male and 114 (43%) were female (M:F = 1.33:1.00). Recurrence was observed in 78 patients (29.3%) of which, 47 were male (30.9%) and 31 were female (27.2%). Based on the initial occurrence, the age for first diagnosis was distributed over the entire age range from 7 to 84 years, and the average was 33.1 years (Fig. 2). The highest incidence was observed in the third decade (78 patients, 29.3%), followed by fourth (63 patients, 23.6%), and second decades (49 patients, 18.4%).

The first occurrence site was analyzed in a total of 274 lesions, including 5 cases of multiple lesions among 266 patients (as shown in Supplemental Table 1). It was found that 93 (34%) lesions occurred in the maxilla, and 181 (66%) lesions occurred in the mandible. The rate of recurrence in the maxilla was 24.7%, and in the mandible 30.9%. By site, 124 lesions occurred in the mandibular ramus, followed by 77 in the maxillary posterior part, and 49 in the maxillary posterior part. More than half of the cases (173, 63.2%) occurred in the posterior region of the mandible and in the ramus. The recurrence rate was highest in the mandibular posterior region (38.7%) and lowest in the maxillary posterior region (24.6%).

Among the 274 lesions, 40.5% was between 3 and 6 crowns (as shown in Supplemental Table 2). The proportions of lesions smaller than 3 crowns in size and larger than 6 crowns were 39.8% and 19.7%, respectively. The recurrence rate increased with increasing size of the lesion, which was statistically significant (p < 0.000). The unilocular form was observed in 68.2%, and the multilocular form was observed in 31.8% of the lesions. In the multilocular form, the recurrence rate was significantly higher than in the unilocular form (p = 0.001).

Enucleation was performed in 203 cases, enucleation following decompression in 67 cases, and en bloc excision in 4 cases (as shown in Supplemental Table 3). The recurrence rate was higher when decompression was performed before enucleation, and it was similar regardless of whether the lesion was unilocular or multilocular.

**Fig. 3:**
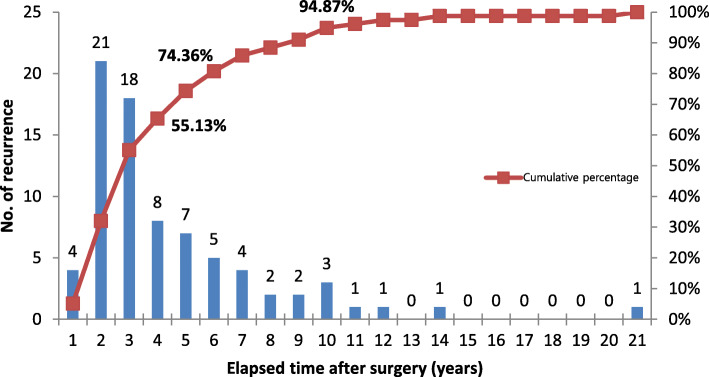
Chronologic recurrence pattern of odontogenic keratocyst

The average recurrence after the first operation was found at 47.5 months (1325 days). The median was 32.5 months (975 days) (Fig. 3). Among all cases of recurrence, 55.1% recurrence occurred within 3 years after surgery, and 74.3% occurred within 5 years. Cross-analysis was performed to identify factors affecting recurrence, but there was no statistical significance in terms of size, location, sex, radiological patterns, and treatment methods. A total of 18 patients experienced more than 1 recurrence, 14 patients had 2 recurrences, and 2 patients had 3 and 4 recurrences.

## Discussion

OKC is a typical developmental cyst with a recurrence rate of 12 to 58.3% [2, 4, 14, 15]. The reasons for this wide range could be differences in the number of cases and the duration of observation, whether lesions with ortho- or para-keratinized epithelium were included, and whether lesions associated with basal cell nevus syndrome were included. The recurrence rate of 28.8% observed in this study was similar to the 30% observed in previous reports using more than 300 cases [16, 17]. Although, the exact reason for the high recurrence rate of OKC has not been established, it is thought to be due to incomplete removal of primary lesion with thin epithelial lining, the presence of satellite cysts, and epithelial remnants.

The average age at initial diagnosis was 33.1 years. While the range was wide (7 to 84 years), patients in the third and fourth decades accounted for almost 53% of all the cases. These results are similar to the results from previous reports of peak incidence between the second and fourth decades of life [18, 19]. In this study, the ratio of rate of incidence in male to female patients was 1.33:1.00. The rate of recurrence in male and female patients was 30.9% and 27.2%, respectively. However, there was no statistically significant difference in the incidence and recurrence rate by sex. Previously, various authors have reported male preponderance [20], no sex difference [21], and female preponderance [22] with reference to occurrence of OKC. Thus, sex-related incidence is controversial.

The primary lesion of OKC occurred in the maxilla in 34% of the cases, and in the mandible in 66% of the cases. A total of 63.2% of lesions occurred in the mandibular ramus and posterior region. Myoung and Hong reported that the recurrence rate in the mandible was higher than in the maxillary lesion [4]. In this study, the recurrence in the mandible was 30.9%, which was higher than the recurrence in the maxilla (24.7%), but there was no statistical significance (p = 0.283). The highest recurrence rate was observed in the posterior mandible (39.7%), and the lowest in the posterior maxilla (24.6%). The differences of recurrence rate between posterior mandible and posterior maxilla were more than 10%, but they were not statistically significant (p = 0.464). The reason for the highest recurrence rate in the posterior mandible is thought to be due to the incomplete resection of the tooth root and the presence of inferior alveolar nerve.

Among the 274 lesions, the size of 3 to 6 crowns was most common (111 lesions), followed by less than 3 crowns (109 lesions), and larger than 6 crowns (54 lesions). In previous reports, the rate of recurrence in relation to size has been controversial. Some researchers reported no correlation [4, 20]. However, in this study, the rate of recurrence increased as the size increased, and this was statistically significant. In addition to the size of the lesion, the rate of recurrence was higher in the multilocular lesions than in the unilocular lesions, which is similar to a previous report [21]. Therefore, if the lesion is multilocular or has a size larger than 6 crowns, a higher rate of recurrence can be predicted, and thus, a long-term follow-up is indicated.

In this study, it was observed that as the size of the lesion increased, the maxillofacial surgeon attempted decompression, followed by enucleation rather than immediately performing enucleation. No recurrence was observed in the cases when en bloc excision was performed. However, in 27.1% of the cases of enucleation and 35.8% of the cases of enucleation after decompression, recurrence was observed. When decompression is performed, the size of the cystic mass decreases, but the surrounding satellite cysts remains. The growth of these satellite cysts is thought to be the reason for higher recurrence [22].

It has been reported that recurrence of OKC occurred after a few decades [3], but many reports have reported that most recurrence occurs within an average of 5 years [2, 19, 23]. In this study, recurrence occurred at 57.5 months on an average. The median was 32.5 months postoperatively, which is similar with the previous study results. In this study, the fastest recurrence was observed about 7 months postoperatively, and the longest recurrence was 21 years after the initial operation. Of all recurring lesions, 74.3% occurred within 5 years after the first operation, and 94.8% occurred within 10 years after surgery. Since about 20% of recurring lesions occurred between 5 years and 10 years postoperatively, periodic follow-up of at least 10 years is indicated following enucleation of OKC. In particular, in the case of a recurrence, there is a tendency for repeated recurrence, so the authors think it is important to build a close follow-up plan.

Many treatment methods have been attempted to reduce this recurrence, such as the Carnoy’s solution [24], cryotherap y[25], and peripheral ostectomy, but no treatment modality, except en bloc excision, has led to a significant reduction in terms of rate of recurrence [16, 17, 26]. Research on target therapy and gene therapy in the future may develop new treatment methods to reduce the occurrence, growth, and recurrence of OKC. Until then, the maxillofacial surgeon should be aware that small-sized OKC is asymptomatic, thus most patients recognize the lesion only when swelling, facial deformation, and infection-related pain occurring due to increased lesion size. In addition, the patient’s tissue deficiency increases as the size of the lesion increases, and consequently, the rate of recurrence increases. Therefore, a regular periodic radiographic examination is necessary to identify the lesion before symptoms occur. The periodic follow-up is important to observe recurrence of OKC, but only few studies have been conducted to prevent recurrence itself. New treatment modalities, which can suppress recurrence, are indicated, such as gene therapy for the PTCH gene, smooth end receptors.

## Conclusion

The average age at which OKC was first diagnosed was 33.1 years, and the male to female ratio was 1.33:1.00. The age group with the highest rate of incidence was the third decade followed by the fourth, second, and fifth decades. The incidence in the maxilla and mandible was 34% and 66%, respectively. The region most commonly involved was the mandibular ramus area. In terms of size, lesions between 3 and 6 crowns were the most common. The rate of recurrence increased as the size of the lesion increased. Enucleation after decompression had higher rate of recurrence (35.8%) than enucleation (27.1%), but there was no statistical significance. The recurrence of OKC was significantly associated with large size, multilocular form, and surgical procedure. A 10-year follow-up period is recommended to determine any recurrence, and a closer follow-up is necessary in cases of recurrence.

## Supplementary Information

**Additional file 1: Supplemental Table 1**. Distribution of occurrence and recurrence site of odontogenic keratocyst. **Supplemental Table 2**. Recurrence rate according to size and radiographic patterns. **Supplemental Table 3**. Recurrence rate according to radiographic patterns and treatment modalities.

## Data Availability

This study was conducted as a retrospective research method, and raw data can be provided at the request of the journal.
