# Supplementary material for: Appropriate follow-up period for odontogenic keratocyst: a retrospective study
Source: Maxillofac Plast Reconstr Surg. 2021 Jul 1;43(1):16. doi: 10.1186/s40902-021-00301-x (PMC8245628; doi:10.1186/s40902-021-00301-x)
Supplement: Supplementary file 1 — Additional file 1: Supplemental Table 1. Distribution of occurrence and recurrence site of odontogenic keratocyst. Supplemental Table 2. Recurrence rate according to size and radiographic patterns. Supplemental Table 3. Recurrence rate according to radiographic patterns and treatment modalities. [file 40902_2021_301_MOESM1_ESM.docx]

**Additional file 1**

**Supplemental Table 1.** Distribution of occurrence and recurrence site of odontogenic keratocyst.

| **Occurrence site** | | **No. of Occurrence (%)** | | **No. of Recurrence (%)** | |
| --- | --- | --- | --- | --- | --- |
| Maxilla | Anterior | 16 | (5.8%) | 4 | (25%) |
|  | Posterior | 77 | (28.1%) | 19 | (24.6%) |
|  | Total | 93 | (34.0%) | 23 | (24.7%) |
| Mandible | Anterior | 8 | (2.9%) | 3 | (37.5%) |
|  | Posterior | 49 | (17.9%) | 19 | (38.7%) |
|  | Ramus | 124 | (45.3%) | 34 | (27.4%) |
|  | Total | 181 | (66.0%) | 56 | (30.9%) |
| Total | | 274 | (100%) | 79 | (28.8%) |

**Supplemental Table 2.** Recurrence rate according to size and radiographic patterns.

|  | | **No. of occurrence (%)** | | **No. of Recurrence (%)** | | ***p*-value** |
| --- | --- | --- | --- | --- | --- | --- |
| Size | < 3 Cr. | 109 | (39.8%) | 21 | (19.3%) | *p* < 0.000 |
|  | 3 to 6 Cr. | 111 | (40.5%) | 32 | (28.8%) |  |
|  | > 6 Cr. | 54 | (19.7%) | 26 | (48.1%) |  |
| Radiol | Uni- | 187 | (68.2%) | 42 | (22.5%) | *p* < 0.005 |
|  | Multi- | 87 | (31.8%) | 37 | (42.5%) |  |
| Total | | 274 | (100%) | 79 | (28.8%) |  |

* Abbreviation: Cr., Crown; Radiol, Radiological finding; Uni-, Unilocular; Multi-, Multilocular

**Supplemental Table 3.** Recurrence rate according to radiographic patterns and treatment modalities.

|  | Unilocular | | | | Multilocular | | | | Total | | | |
| --- | --- | --- | --- | --- | --- | --- | --- | --- | --- | --- | --- | --- |
|  | No. | Rec (%) | | p-value | No. | Rec (%) | | p-value | No. (%) | | Rec (%) | |
| Enuc | 144 | 29 | (20.1%) | 0.164 | 59 | 26 | (44.0%) | 0.883 | 203 | (74.1%) | 55 | (27.1%) |
| Dec-Enuc | 43 | 13 | (30.2%) |  | 24 | 11 | (45.8%) |  | 67 | (24.4%) | 24 | (35.8%) |
| En bloc | - | | |  | 4 | 0 | (0%) |  | 4 | (1.5%) | 0 | (0%) |
| Total | 187 | 42 | (22.5%) |  | 87 | 37 | (42.5%) |  | 274 | (100%) | 79 | (28.8%) |

* Abbreviation: Enuc, Enucleation; Dec-Enuc, Enucleation following decompression; En bloc, En block excision; Rec, Number of recurrence
